# Transient Expression of *Candidatus* Liberibacter Asiaticus Effector Induces Cell Death in *Nicotiana benthamiana*

**DOI:** 10.3389/fpls.2016.00982

**Published:** 2016-07-06

**Authors:** Marco Pitino, Cheryl M. Armstrong, Liliana M. Cano, Yongping Duan

**Affiliations:** ^1^U.S. Horticultural Research Laboratory, Agricultural Research Service, United States Department of AgricultureFort Pierce, FL, USA; ^2^Institute of Food and Agricultural Sciences, Department of Plant Pathology, Indian River Research and Education Center, University of FloridaFort Pierce, FL, USA

**Keywords:** huanglongbing, *Candidatus* Liberibacter asiaticus, bacterial effectors, cell death, *Nicotiana benthamiana*, callose deposition, chloroplast localization

## Abstract

*Candidatus* Liberibacter asiaticus “Las” is a phloem-limited bacterial plant pathogen, and the most prevalent species of Liberibacter associated with citrus huanglongbing (HLB), a devastating disease of citrus worldwide. Although, the complete sequence of the Las genome provides the basis for studying functional genomics of Las and molecular mechanisms of Las-plant interactions, the functional characterization of Las effectors remains a slow process since remains to be cultured. Like other plant pathogens, Las may deliver effector proteins into host cells and modulate a variety of host cellular functions for their infection progression. In this study, we identified 16 putative Las effectors via bioinformatics, and transiently expressed them in *Nicotiana benthamiana*. Diverse subcellular localization with different shapes and aggregation patterns of the effector candidates were revealed by UV- microscopy after transient expression in leaf tissue. Intriguingly, one of the 16 candidates, Las5315mp (mature protein), was localized in the chloroplast and induced cell death at 3 days post inoculation (dpi) in *N. benthamiana*. Moreover, Las5315mp induced strong callose deposition in plant cells. This study provides new insights into the localizations and potential roles of these Las effectors *in planta*.

## Introduction

Citrus huanglongbing (HLB), also known as citrus greening, is a devastating disease with high economical costs to the worldwide citrus industry (Hodges and Spreen, 2012). The disease is caused by three species of alpha-proteobacterium, “*Candidatus* Liberibacter asiaticus (Las),” “*Ca*. L. africanus,” and “*Ca*. L. americanus.” Las, the most widespread pathogen, is vectored by the Asian citrus psyllid (ACP) *Diaphorina citri Kuwayama* (Hemiptera: Psyllidae) (Jagoueix et al., 1996; Garnier et al., 2000; Bove, 2006; Hall et al., 2013). Las attacks all species and hybrids in the *Citrus* genus, and upon infection, Las resides in the phloem of the host, which causes a systemic disease and can eventually result in the death of the tree (Halbert and Manjunath, 2004; Bove, 2006; Gottwald, 2010). Once a tree is infected, it is extremely difficult to cure, and currently there is no adequate strategy for HLB management.

Host-pathogen relationships encompass a myriad of protein-protein interactions that include not only pathogen detection and removal by the host but also mechanisms to avoid such processes by the pathogen. Understanding this interplay would open the door to more successful HLB control methods, however, the corresponding mechanisms are currently largely unknown. Plants, including citrus, are able to detect conserved microbial molecular signatures termed pathogen-associated molecular patterns (PAMP), and initiate a process known as PAMP-triggered immunity (PTI) (Boller and Felix, 2009; Segonzac and Zipfel, 2011). Pathogens, in turn, can suppress the PTI response by deploying diverse effector proteins that modulate various host cellular functions in order to promote bacterial colonization and replication (Chisholm et al., 2006; Hogenhout et al., 2009; Dodds and Rathjen, 2010). Effectors that are deployed to suppress host defenses may be recognized by plant disease resistance (R) proteins in particular host genotypes, resulting in effector-triggered immunity (ETI) (Jones and Dangl, 2006; Boller and Felix, 2009). A common strategy for plant pathogens to avoid the immune responses is to either lose their effectors or mutate by developing new effectors that are once again able to suppress ETI (Stergiopoulos and De Wit, 2009). This is important because together PTI and/or ETI limit microbial entry, restrict pathogen propagation, or kill pathogens inside plant tissues (Dou and Zhou, 2012). Overall, an in-depth investigation into the roles of putative Las candidate effectors is key not only to understanding how Las manipulates host defense responses and results in HLB disease but to identifying pathogenicity mechanisms that can ultimately lead to the development of novel control methods.

In 2009, the complete genome sequence of Las was obtained (Duan et al., 2009), enabling heterologous expression of Las proteins to be performed. Expression studies have demonstrated that the Las bacterium can act as an “energy parasite” through its encoded ATP translocase, which allows for direct ATP/ADP importation from its host cells (Vahling et al., 2010), and that two novel autotransporter proteins encoded in the Las prophages target the mitochondria of plants (Hao et al., 2013). In addition, using *Liberibacter crescens* strain BT-1 as model system, a peroxidase of Las was confirmed to be involved in suppressing the plant innate immunity via detoxification of H_2_O_2_ (Jain et al., 2015). From the host plant aspect, Las infection is correlated with significant changes in protein regulation in *Citrus paradisi*, indicating that Las actively alters molecular processes in citrus (Nwugo et al., 2013).

Unfortunately, even with the examination of citrus transcriptomes in response to Las infection and comparative transcriptome analyses (Albrecht and Bowman, 2008, 2012; Kim et al., 2008; Rawat et al., 2015; Zhong et al., 2015), Las genes involved in psyllid or citrus colonization remain largely unknown. This lack of accomplishment is due in part to the limited success in culturing the bacterium (Sechler et al., 2009) and the large number of hypothetical proteins in the Las genome (Duan et al., 2009). Alternative tactics involving heterologous systems are necessary in order to overcome these hurdles and speed the process of identification and functional characterization of candidate Las effector proteins (Boller and Felix, 2009; Segonzac and Zipfel, 2011; Dou and Zhou, 2012). Genome sequencing together with computational methods have revealed a wide catalog of candidate effectors genes in filamentous plant pathogenic: fungi (Chaudhari et al., 2014; Petre and Kamoun, 2014), oomycetes (Ellis et al., 2009), nematodes (Davis et al., 2008), bacteria (Block et al., 2008; Zhou and Chai, 2008; Hogenhout et al., 2009), and insects such as aphids (Bos et al., 2010; Pitino and Hogenhout, 2013). To functionally characterize pathogen effectors and how these proteins manipulate host cells, it is crucial to identify the host compartments where the effectors localize (Alfano, 2009). Thus, subcellular localization is one of the first aspects to consider when assessing effector function.

Utilizing a pathogen secretome analysis we uncovered 16 putative effectors in the Las genome. Similar to other studies, in an attempt to characterize these candidate effector proteins, they were transiently expressed in *N. benthamiana* leaves to allow both the localization pattern as well as any overt phenotype to be observed (Vleeshouwers et al., 2008; Wang et al., 2011; Du et al., 2014; Petre et al., 2015). These findings not only help establish *N. benthamiana* as a valuable heterologous system for fast-forward effectoromic analysis of plant pathogens regardless of their host plant (Petre et al., 2016) but also represent the first attempt to characterize and locate putative Las effectors by combining a pathogen secretome analysis with an *in planta* transient expression assay. Knowledge obtained from this Las effector screen enhances our understanding of HLB and may aid in the development of new and more efficient management strategies.

## Materials and methods

### Genomic DNA extraction of Las bacterial pathogen

In addition to transmission by insects, Las can be experimentally transferred to non-rutaceous hosts such as periwinkle (*Catharanthus roseus*), which acts as a model organism for HLB because when infected via parasitic dodder (*Cuscuta campestri*) it allows Las to replicate to high titers (Garnier and Bové, 1983). Total genomic DNA was extracted from Las infected periwinkle leaves using the following protocol. Collected leaf tissue was chopped via razorblade and transferred to an autoclaved 2 ml screw-capped tube containing two 4 mm silicone-carbide sharp particles and four 2.3 mm chrome-steel beads in 800 μL of extraction buffer (100 mM HCl, 30 mM EDTA, 500 mM NaCl, 5% polyvinylpyrrolidone and 3% CTAB). Tissue was homogenized using a Fast Prep®-24 homogenizer at speed 6.0 m/s for 60 s and incubated at 65°C for 30 min after the addition of 80 μl 20% SDS. After adding one-third volume of 5 M potassium acetate, the tubes were incubated on ice for 5 min and centrifuged at 18,000 rpm (Eppendorf 5424, Sunnyvale, CA, USA) for 5 min to remove plant debris. The supernatants were removed and placed into a new tube and centrifuged for an additional 10 min at the same speed and then 800 μL of supernatant was transferred to a new 1.5 ml tube containing two-third volume of cold isopropanol. The sample was then placed in a Genesee filter column (Genesee Scientific, San Diego, CA), centrifuged 1 min at 10000 rpm and washed twice with 70% ethanol. Samples were then eluted with 100 μl nuclease-free water and analyzed using the Nanodrop 1000 spectrophotometer (Thermo Scientific, Wilmington, DE) with the concentrations being adjusted to 50 ng/μl DNA prior to PCR.

### Expression of *Las5315* effector gene from infected and uninfected citrus by RT-PCR

Total RNA was extracted from Las infected and uninfected *Citrus paradise* (Grapefruit cultivar Duncan) and *Citrus sinensis* (Sweet orange cultivar Valencia) leaves using TRIzol reagent according to the manufacturer's protocol. DNA contaminations were removed by treating RNA extraction with RNase-free DNase (QIAGEN, West Sussex, UK) and subsequently purified with QIAamp columns (QIAGEN). First-strand cDNA was synthesized at 42°C from total RNA using M-MLV (Invitrogen) reverse transcriptase according to the manufacturer's instructions with the individual PCR reactions containing 1 μl of cDNA, 0.5 μl of each specific primer (10 pmol/μl) (Table S1), 10 μl of 2X Green GoTaq® Reaction Buffer (Promega) in a final volume of 20 μl. The following standard thermal profile was used for all amplifications: 95°C for 3 min followed by 30 cycles of 95°C for 30 s, 58°C for 30 s, and 70°C for 30 s.

### Bioinformatic identification of Las secreted effector candidates

We used as input the genome version v1 from Las isolate psy62 (Duan et al., 2009) (genbank accession NC_012985.3), which is also deposited under accession 537021.9 at Pathosystems Resource Integration Center (PATRIC) (https://www.patricbrc.org/portal/) (Wattam et al., 2014). A protein was predicted to be secreted when a signal peptide was present using SignalP v2.0 and (Nielsen et al., 1997), signalP4.0 and no transmembrane domains were present using TMHMM v2 (Petersen et al., 2011). Then, putative Las secreted candidate effectors were screened for: (1) secreted proteins that were ≤250 amino acids and (2) secreted proteins with a previously uncharacterized function according to the NCBI BLAST database. The genbank “GI” accessions for all 16 secreted candidate effectors are shown in Table 1.

**Table 1:** **Features of 16 Las candidate secreted effectors proteins**.

**Gene ID v1 (genbank accession)**	**Sequence length (aa)**	**Number of cysteines**	**Chloroplast target domain**	**Subcellular localization in *Nicotiana benthamiana***
CLIBASIA_00460 (ACT56680.1; GI:254039884)	98	2	NO	Long shape aggregates in the cytoplasm
CLIBASIA_00525 (ACT56693.1; GI:254039897)	97	1	NO	Uninformative and unspecific
CLIBASIA_00530 (ACT56694.1: GI:254039898)	97	1	NO	Round big vesicles in the cytoplasm
CLIBASIA_02215 (ACT57029.1; GI:254040233)	120	1	NO	Aggregates in the cytoplasm
CLIBASIA_02470 (ACT57080.1; GI:254040284)	131	7	NO	Small vesicles in the cytoplasm
CLIBASIA_03230 (ACT57232.1; GI:254040436)	162	7	NO	Aggregates in the cytoplasm
CLIBASIA_03695 (ACT57317.1; GI:254040521)	113	5	NO	Vesicles in the cytoplasm
CLIBASIA_04025 (ACT57379.1; GI:254040583)	96	1	NO	Vesicles in the cytoplasm
CLIBASIA_04040 (ACT57382.1; GI:254040586)	159	1	NO	Small vesicles in the cytoplasm
CLIBASIA_04320 (ACT57436.1; GI:254040640)	215	2	NO	Aggregates and dots in the cytoplasm
CLIBASIA_04330 (ACT57438.1; GI:254040642)	229	4	NO	Cytosolic bodies in the cytoplasm
CLIBASIA_04425 (ACT57457.1; GI:254040661)	125	2	NO	Strong signal with variable shape in the cytoplasm
CLIBASIA_04560 (ACT57483.1; GI:254040687)	195	3	NO	Vesicles in the cytoplasm and nucleus distribution
CLIBASIA_05115 (ACT57594.1; GI:254040798)	185	2	NO	Aggregates in the cytoplasm
CLIBASIA_05320 (ACT57630.1; GI:254040834)	85	1	NO	Punctuated distribution within vesicles in the cytoplasm
CLIBASIA_05315 (ACT57629.1; GI:254040833)	154	2	YES	Chloroplast within the cytoplasm

### Amplification and cloning of Las secreted effector candidates

The mature form of each effector protein without its corresponding signal peptide was cloned to create a C-terminal translation fusion with green fluorescent protein (GFP) under the control of 35S promoter in the binary vector ImpGW405 (Nakagawa et al., 2007). Primers were designed to amplify the corresponding ORFs and contained the complete ATTB sites (Table S1). The sequences were amplified using Las infected periwinkle DNA and HIFI PCR mastermix (Clontech, Mountain View, CA, USA). The PCR fragments were first cloned into pDONRzeo by Gateway® BP Clonase® II (Invitrogen, Carlsbad, CA, USA) and then subcloned into the Gateway destination vectors ImpGWB405 (Nakagawa et al., 2007).

### Transient expression of Las secreted effectors in *Nicotiana benthamiana*

Sequences encoding for mature protein form (lacking signal peptide) of a total of 16 effector candidates were cloned into the binary vector ImpGWB405 and transformed into *Agrobacterium tumefaciens* strain GV3101. Agrobacterium transformant cells were cultured overnight in LB medium with 50 μg ml^−1^ of rifampicin and 100 μg ml^−1^ spectinomycin and resuspended in 10 mM MgCl_2_. The culture was diluted to an optical density at 600 nm (OD_600_) of 0.5 and acetosyringone (final concentration of 100 μM) was added. For each construct, we infiltrated three leaves of young *N. benthamiana* plants with the *A. tumefaciens* suspension. Agroinfiltrated plants were kept in a greenhouse for the duration of the experiment. The agrobacteria-infiltrated leaves were observed for phenotypic changes and then detached from plants and analyzed under microscope (Olympus BX51-P, Center Valley, PA, USA) equipped with a UV light source at 3 dpi for protein localization.

### Staining of callose deposition and relative callose intensity measurement

Aniline blue staining allowed visualization of callose through fluorescence microscopy. An aniline blue signal indicates deposited callose. Four *N. benthamiana* leaves were used after 1, 2, 3, and 4 dpi for Las5315mp and the GFP control. To distain the chlorophyll, ethanol (90% v/v) was added for 6 h at 37°C, followed by ethanol (50% v/v) for 1 h, ethanol (30% v/v) for 1 h, and finally dH_2_O for 2 h. The distained leaves were incubated for 2 h in 150 mM K_2_HPO_4_ and 0.01% aniline blue, samples were embedded in 50% glycerol before microscope analysis. Three leaves from 3 different plants were used in the analysis.

Callose was quantified from digital photographs by the number of white pixels (callose intensity) relative to the total number of pixels covering plant material, using Photoshop CS5 software (Adobe System, San Jose, CA, USA). Callose was selected automatically, using the “Color Range” tool. Data sets were analyzed for statistical differences by Student's *t*-tests (^*^P < 0.01).

### H_2_O_2_ production and electrolyte leakage measurement

H_2_O_2_ detection was conducted as described in previous studies with minor modification (Zhang et al., 2001; Liao et al., 2013). Briefly, *N. benthamiana* leaf discs expressing Las5315mp were collected using a circular 4 mm diameter cork borer at 4 dpi. 6 leaf discs from three leaves were placed into a loading buffer with 50 mM Tris-KCl (pH 7.2) containing 100 μM of H2DCF-DA for 10 min. Fluorescence emission was measured by the LUMIstar microplate luminometer (excitation wavelength, 484 nm; emission wavelength, 525 nm). To quantify cell death, electrolyte leakage was performed according to (Huang et al., 2013). Five leaf discs (~5 mm in diameter) were collected from the Las5315mp and control *Agrobacterium*-infiltrated areas and immersed in 10 mL of non-ionic, double-distilled water. After incubation at room temperature for 1 h with shaking at 160 rpm, conductivity of the solution was measured using a conductivity meter (Horiba scientific, Edison, NJ, USA).

## Results

### Sixteen putative las effectors were selected

**Figure 1:**
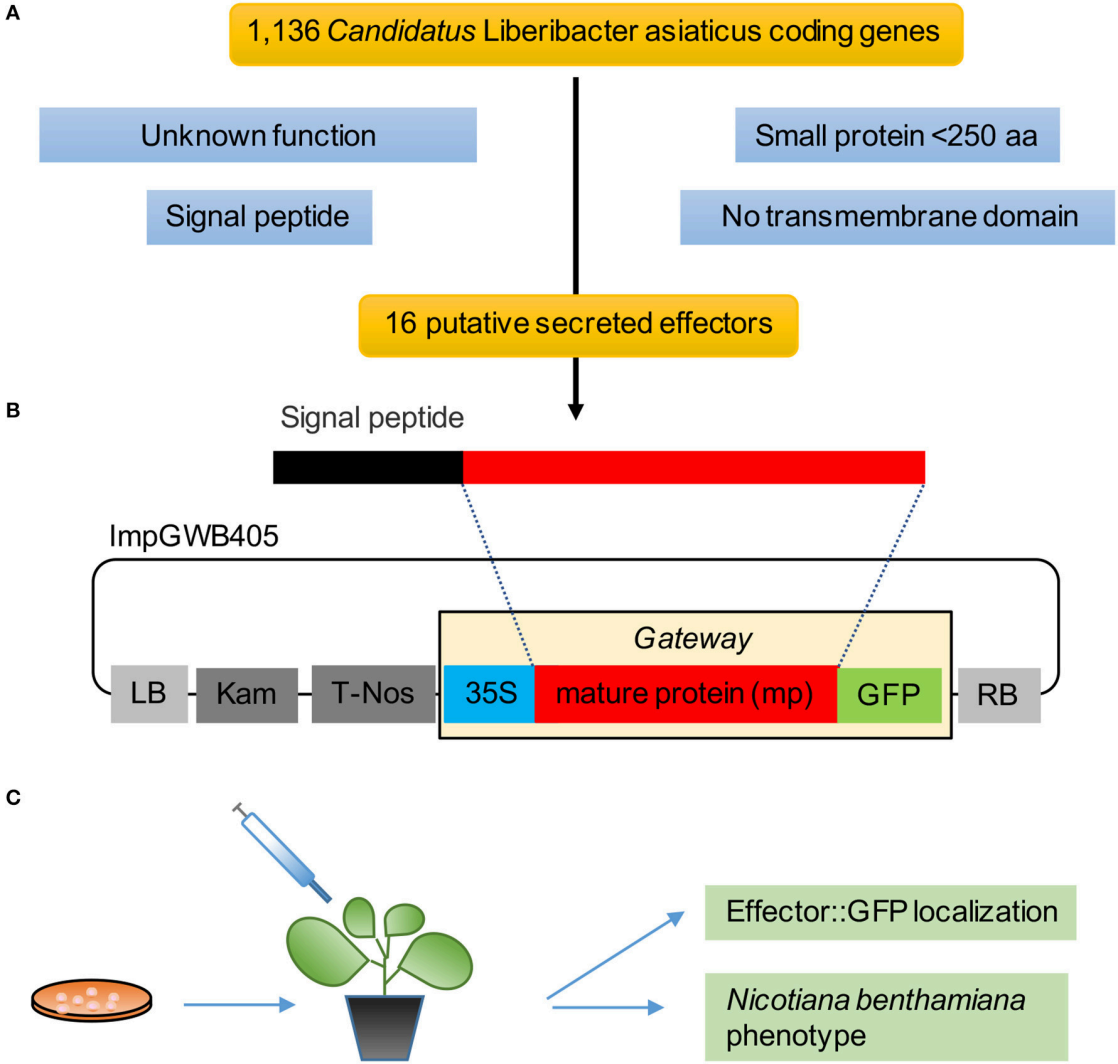
**Pipeline to identify Las effectors. (A)** Selection criteria for Las effectors yielded 16 putative genes from the 1,136 CDS in the Las genome. **(B)** Mature protein sequences were cloned in the gateway binary vector ImpGWB405. These clones were transformed into *Agrobacterium tumefaciens* strain GV3101 for transient expression in *N. benthamiana* leaves. **(C)** Functional analyses included GFP localization and *in planta* phenotype.

We analyzed 1,136 protein-encoding genes of Las Psy62 genome version 1 (genbank accession NC_012985.3) (Duan et al., 2009) for the presence of signal peptide and lack of transmembrane domain. To identify candidate effectors, we filtered the secretome for (1) proteins ≤250 amino acids in length and (2) previously uncharacterized proteins (Figure 1A). These 16 secreted effector proteins exhibit sequence lengths that varied from 85 to 229 amino acids. The mature proteins (Figure 1B), without their putative signal peptides, were transiently expressed in *N. benthamiana* in order to characterize the location of the putative effectors and analyze phenotypic changes produced by the proteins (Figure 1C).

### Las candidate effectors exhibited diverse subcellular localization patterns in *N. benthamiana*

**Figure 2:**
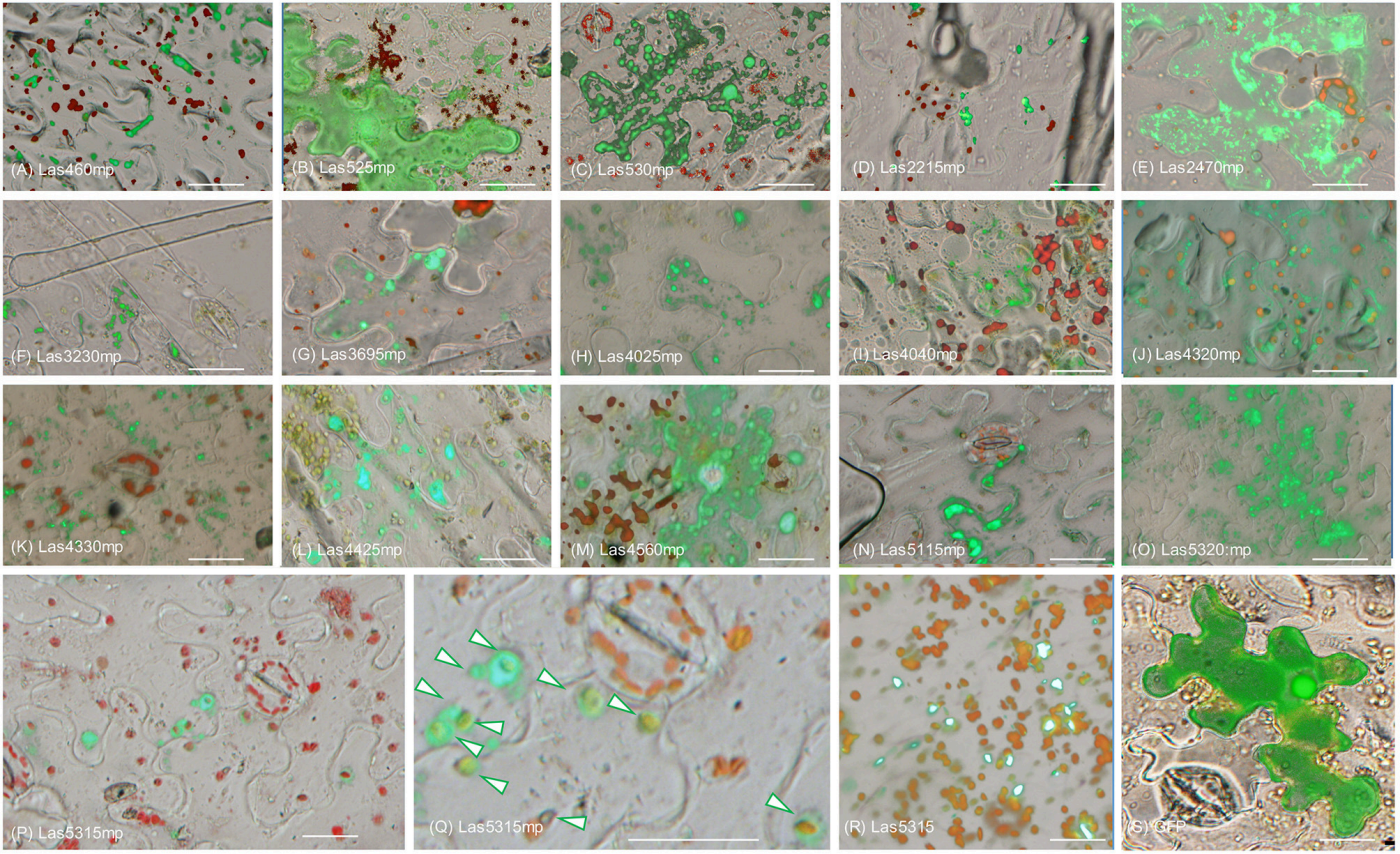
**Subcellular localization of putative Las effector proteins in ***Nicotiana benthamiana*****. Epi-fluorescence micrograph overlaid with bright field micrograph of each of the 16 effector translation fusions expressed in *N. benthamiana*. Images depict protein effector**:** GFP (green) and chlorophyll autoflourescence (red). **(A–Q)** Micrograph of the localization of the 15 candidate effectors that did not elicit a phenotype in *N. benthamiana*. **(P)** Image shows localization of Las5315mp:GFP (green) surrounding chloroplasts (red). **(Q)** Enlarged image of panel **(R)** with arrows indicating Las5315mp localization surrounding chloroplasts. **(R)** Las5315::GFP accumulated in aggregate structures **(S)** GFP only. Scale bars are 20 μm.

In order to determine the location of the 16 putative effectors in the plant cells, C-terminal translational fusions were produced using the mature form of each protein and the green-fluorescent protein (GFP) expressed from the 35S promoter in a gateway binary vector (Figure 1B). The leaf epidermal layer of the infiltrated zones for each of the 16 candidate effectors showed different localization and accumulation patterns in the plant cells (Figure 2, Figure S1) compared to the control GFP only (Figure 2S). In particular, Las460mp was distributed in the cytosol and accumulated in small vesicles and in elongated shapes. Las525mp was dispersed throughout the cytosol and the nucleus. Las530mp accumulated heavily in vesicle structures while Las2215mp accumulated in aggregated structures. Las2470mp had an intense signal with dot shape bodies covering the total cell area. Las3230mp formed small crystal shape aggregates, while Las3695mp showed an increased signal intensity and was localized in big vesicles, and Las4025mp accumulated in small vesicles and cytosol bodies. Las4040mp was similar to Las4025mp with small vesicles and punctate spots, Las4320mp was expressed evenly in the cell taking the shape of dots, Las4330mp small cytosolic bodies, Las4425mp accumulate in large cytosolic aggregates, Las4560mp was localized in large and small bodies and in the nucleus, Las5115mp accumulated mostly on the edge of the cell with large aggregates, and Las5320mp fluoresced strongly and accumulated in dots inside vesicles. The Las5315mp effector protein was localized in vesicles surrounding chloroplast (Figures 2P,Q), while the effector Las5315 that contained the signal peptide sequence was not present in chloroplast, but accumulated as crystal shape aggregates (Figure 2R).

### Las5315mp induced cell death and is associated with H_2_O_2_ accumulation, electrolyte leakage, and callose deposition in *N. benthamiana*

**Figure 3:**
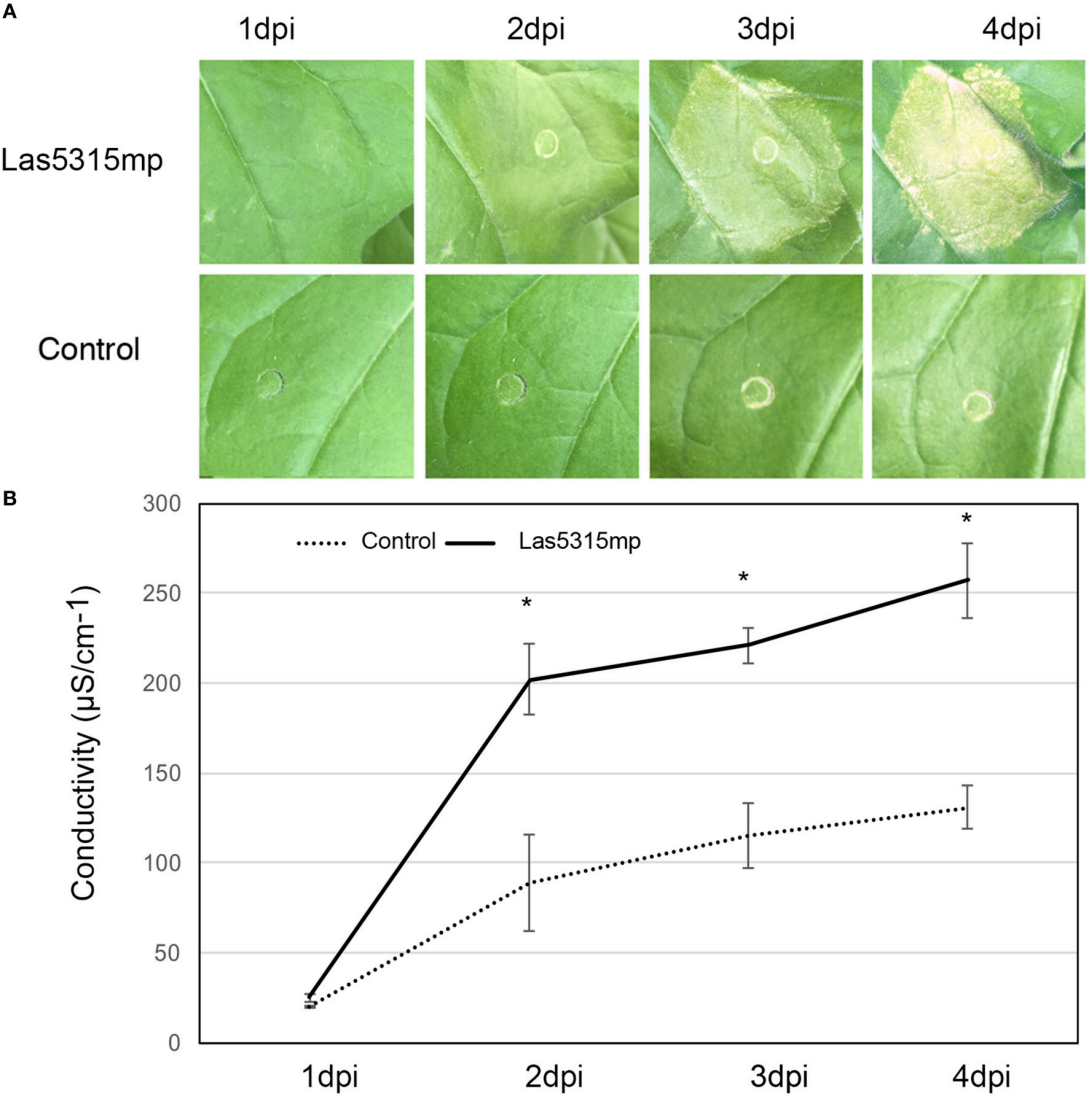
**Cell death triggered by Las5315mp results in ion leakage**. **(A)** Progression of cell death in *N. benthamiana* leaves infiltrated with Las5315mp. Example of leaves taken for electrolyte leakage. Photographs were taken at 1, 2, 3, and 4 dpi (dpi: days post infiltration). **(B)** Electrolyte leakage was measured in the infiltrated leaves at the indicated time points after agroinfiltration each data point represent the average + SD from 5 infiltrated leaves. The experiments were repeated three times with similar results. Asterisks signs indicate statistically significant differences from the control as calculated by Student's *t*-test (^*^P < 0.01).

**Figure 4:**
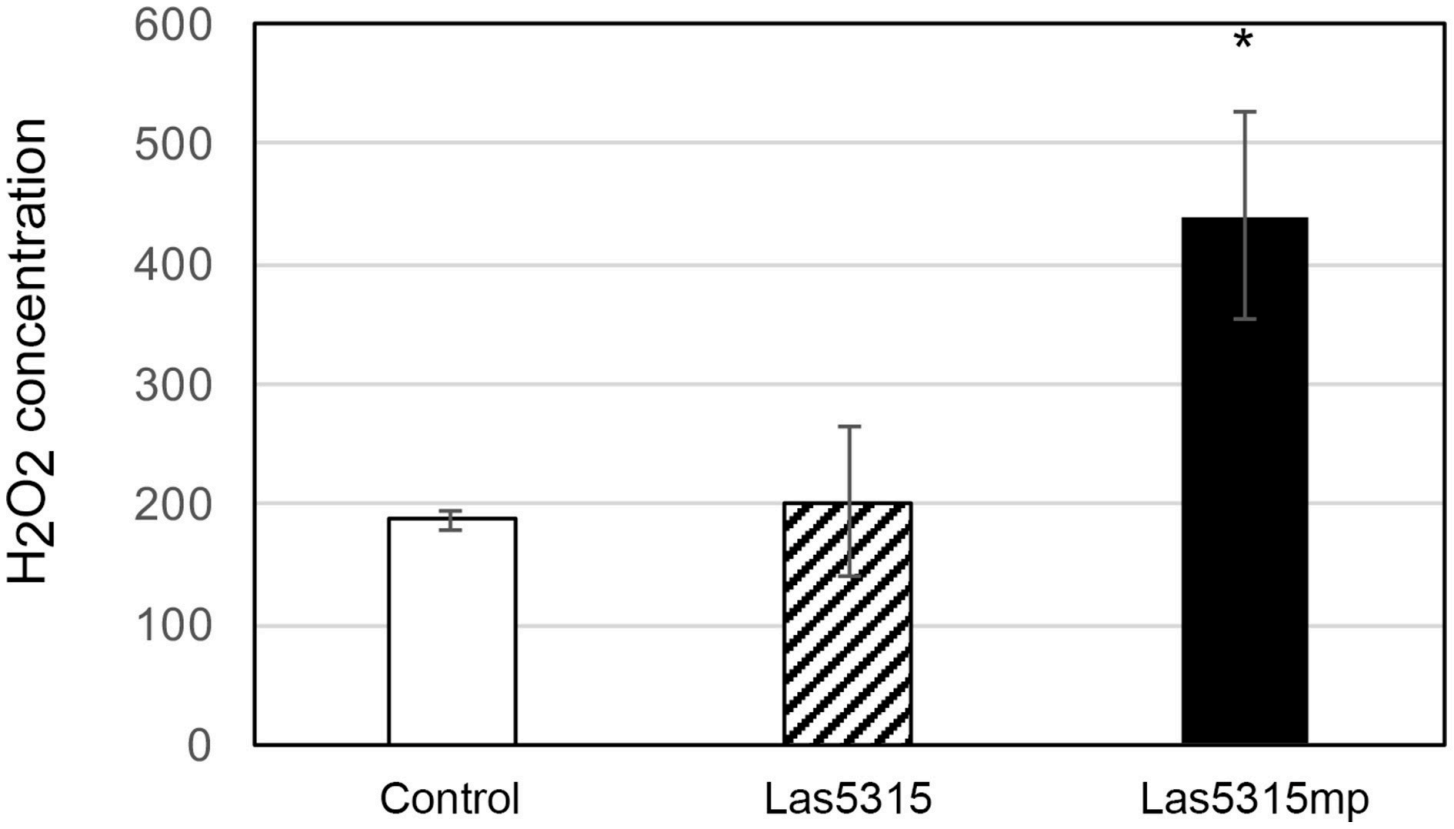
**Cell death triggered by Las5315mp results in the accumulation of H_**2**_O_**2**_**. The concentration of H_2_O_2_ was determined in leaves agro-infiltrated with the Las5315mp effector or the empty vector (EV) using H_2_DCFDA as an indicator at 4 dpi. Leaves were used as a control. Error bars represent the standard deviation (*n* = 8). Asterisks signs indicate statistically significant differences from the control as calculated by Student's *t*-test (^*^P < 0.01).

In an effort to further characterize these effectors and define a potential role regarding Las virulence, putative effector genes were transiently expressed in *N. benthamiana* and their phenotypes were observed. Of the 16 putative effectors only Las5315mp appeared to induce necrosis or cell death (Figure 3A and Figures S2A–C). Compared to the negative control *at 3 dpi*, cell death appeared in the *N. benthamiana* leaf infiltrated with the mature form of effector Las5315mp at three different concentrations: OD_600_ = 0.1, 0.3 and 0.5 (Figure S2A). The cell death phenotype was not observed in the premature form containing the signal peptide, Las5315, at the same three concentrations (Figure S2B). A side-by-side comparison on the same leaf demonstrated that chlorosis and increasing levels of cell death could be observed at 7 dpi in the infiltration zone expressing Las5315mp, while visually Las5315 remained unchanged (Figure S2C). Cell death is often associated with electrolyte leakage resulting from membrane damage (Bai et al., 2012). Ion leakage assays were performed to quantify the cell death induced by Las5315mp. As shown in (Figure 3B) significant amounts of ion leakage were detected 2, 3, and 4 days dpi after infiltration with the *Agrobacterium* harboring the *Las5315mp*. The cell death produced upon expression of Las5315mp is associated with H_2_O_2_ accumulation in the leaves (Figure 4) and electrolyte leakage (Figure 3B). Ion leakage was not induced by agroinfiltration of the control. These data confirm the cell death activity of Las5315mp.

**Figure 5:**
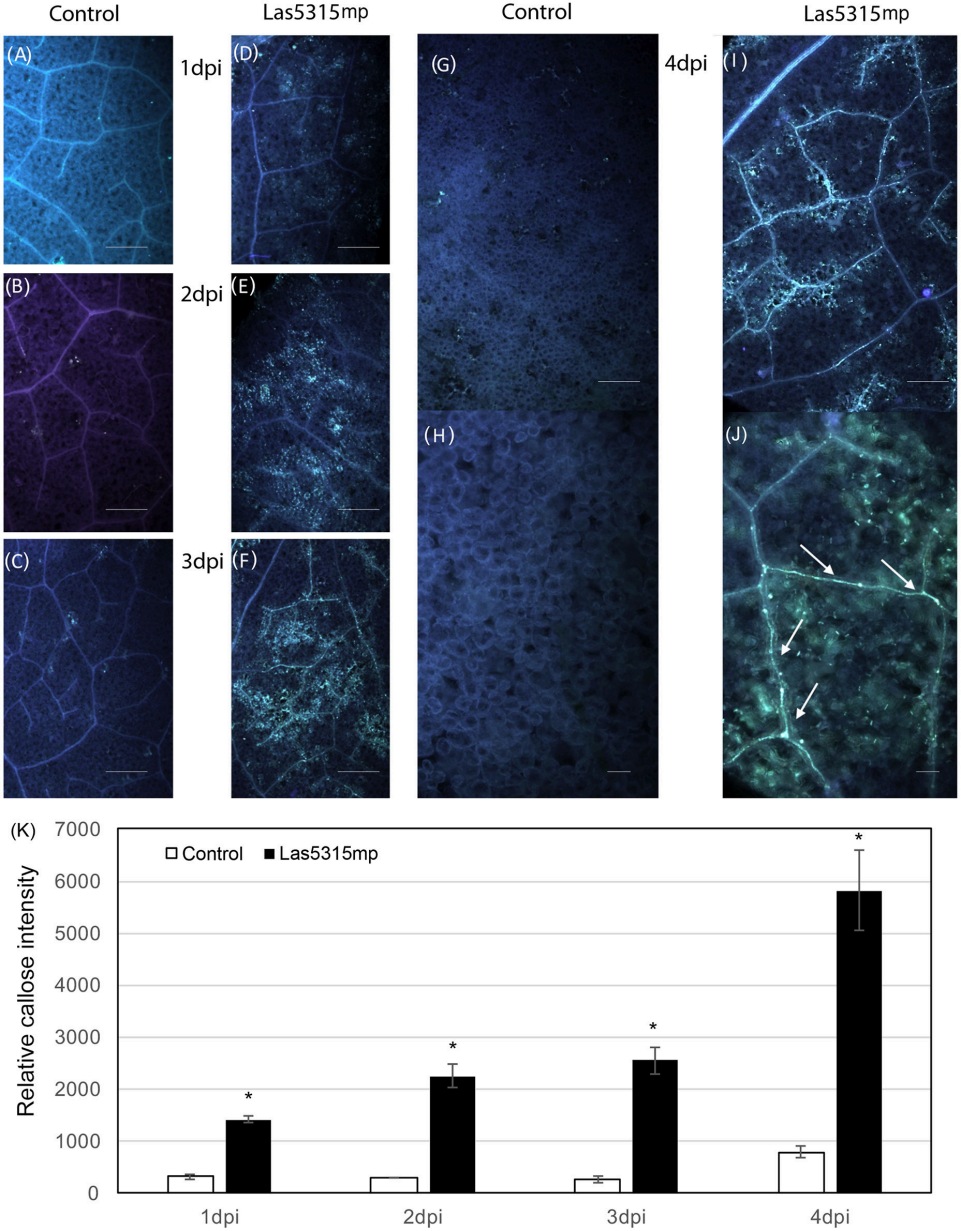
**Callose deposition after Las5315mp agroinfiltration**. *N. benthamiana* leaves agroinfiltrated with either the GFP control **(A–C,G)** or with Las5315mp **(D–F,I)** corresponding to 1, 2, 3, and 4 dpi respectively. Enlarged image of the leaf at 4 dpi infiltrated with either GFP **(H)** or Las5315mp **(J)** showing accumulation of callose in the vascular tissue (arrows). Leaves were stained with aniline blue and callose deposition was observed under a UV epifluorescence microscope. **(K)** Relative callose deposition was measured in the infiltrated leaves at the indicated time points after agroinfiltration (dpi: days post infiltration). Asterisks signs indicate statistically significant differences from the control as calculated by Student's *t*-test (^*^P < 0.01).

In addition to ion leakage and H_2_O_2_ accumulation, fluorescence microscopy with aniline blue staining revealed a strong callose deposition induced by Las5315mp from 1 dpi (Figure 5). This accumulation continued to increases both within the plant cells and inside the vascular tissue for the entire 4 day observation period.

### Las5315 contained a signal peptide and a chloroplast targeting domain

**Figure 6:**
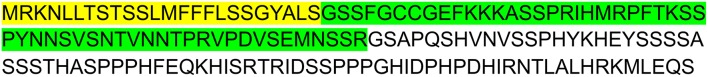
**Las5315mp secreted effector sequence and its predicted C-terminal chloroplast targeting domain**. Amino acid sequence of Las5315mp with N-terminal signal peptide (highlighted in yellow) predicted using SignalP v4 (Petersen et al., 2011), the cleavage site between 24 and 25 amino acid positions (ALS-GS), and the chloroplast targeting (highlighted in green) predicted using ChloroP v1.1 (Emanuelsson et al., 1999).

Sequence analysis of the Las5315mp revealed a 154 aa protein with a signal peptide predicted using SignalP v2.0 (Nielsen et al., 1997) and SignalP v4.0 (Petersen et al., 2011), a cleavage site between position 24 and 25 and no transmembrane domains using TMHMM v2 (Krogh et al., 2001) (Figure 6). Downstream of the N-terminal signal peptide sequence of Las5315 effector protein we found a chloroplast targeting peptide domain of 56 aa length (Figure 6) using ChloroP 1.1 (Emanuelsson et al., 1999) with a score of 0.562 and CS-score of 3.203.

### *Las5315* gene is expressed in Las-infected citrus

In order to determine if *Las5315* is indeed expressed in citrus infected with Las, reverse transcription PCR was used to identify the level of expression of *Las5315* in both Las infected and uninfected grapefruit and sweet orange. Amplicons produced via reverse transcription PCR demonstrate the expression of *Las5315* both grapefruit and sweet orange but only when infected with Las.

## Discussion

“*Ca*. Liberibacter asiaticus” is an insect-vectored obligate intracellular bacterium with a significantly reduced genome associated with HLB disease in citrus. Powerful functional genomic and genetic approaches integrated with comparative studies have contributed a great deal to the knowledge and identification of global gene expression changes during HLB disease development (Albrecht and Bowman, 2008, 2012; Fan et al., 2011; Martinelli et al., 2012, 2013; Aritua et al., 2013; Mafra et al., 2013; Rawat et al., 2015). Other studies have revealed the disruption in carbohydrate source and phloem plugging as main causes of HLB symptoms (Liao and Burns, 2012; Martinelli et al., 2013). However, knowing how effector proteins function in host plant cells is key to fully understanding the molecular basis behind the pathogen-plant interactions and for the development of sustainable management strategies.

Las, like spiroplasmas and phytoplasmas, is injected directly into the cytoplasm of phloem cells by its insect vector. It is the intracellular nature of these organisms that allows them to secrete proteins into the host cell cytoplasm despite their lack of type III secretion systems, permitting movement of these proteins to other plant cells via plasmodesmata (Hogenhout and Loria, 2008). Several examples of his include the plant gene-regulating effector, SAP11, which contains a nuclear localization signal that is functional in plant cells (Hogenhout et al., 2008) and SAP54, which manipulates its host to produce leaf-like flowers, making it more attractive for colonization by phytoplasma leafhopper vectors (Maclean et al., 2014). Las appears to contain all type I secretion system genes necessary for both multidrug efflux and toxin effector secretion. Moreover, all proteins required for the first step of the type II secretion system, the general secretory pathway (TC 3.A.5.) responsible for the export of proteins to the periplasm (Pugsley, 1993), were found in the “*Ca*. L. asiaticus” Psy62 genome (Duan et al., 2009). In addition, 10 putative proteins required for pilin secretion and assembly as part of the main terminal branch MTB (TC 3.A.15) were found, indicating the potential for a type IV pilus secretion assembly (Duan et al., 2009).

Therefore, in an attempt to identify novel proteins that function in the pathogenesis of HLB, we analyzed and selected the most promising candidate effector proteins from the current reference Las genome Psy62 (Duan et al., 2009). Based on the assumption that Las secretes proteins into the plant to manipulate host processes and facilitate colonization, proteins with a predicted signal peptide that did not contain transmembrane domains and had no predicted function were selected for further study. We obtained 16 candidates using this criteria and created C-terminal translation fusions for localization studies in plants by cloning the mature form of each candidate (sequence without the signal peptide regions) into the GFP vector ImPgwb405 (Figures 1A,B).

Pathogen effectors are known to target different parts of the host cell including nuclear components, membranes, and subcellular compartments such as the mitochondria or chloroplasts. This targeting of cellular components is a broadly conserved pathogenic strategy that controls the fate of cell organelles and regulates cellular activities (Deslandes and Rivas, 2012; Lindeberg et al., 2012). Mitochondria for instance play important roles in biological processes such as energy conversion for ATP synthesis, ion homeostasis, and calcium storage as well as in plant innate immunity signaling (Maxwell et al., 2002). Effector proteins produced by pathogens, such as HopG1 from *Pseudomonas syringe*, suppress the host's innate immunity by disrupting mitochondrial function (Block et al., 2010). The mitochondria is not the only system targeted by *P. syringae*, which produces the effectors HopI1, HopN1, HopK1, and AvrRps4 that localize to chloroplasts instead. Hopl1 and HopN1 are known to inhibit photosystem II (PSII) activity in chloroplast preparations by degrading PbsQ (Rodríguez-Herva et al., 2012) while HopK1 and AvrRps4 suppress plant immunity (Li et al., 2014).

Although only one effector displayed a clear cell death phenotype in *N. benthamiana*, all of the putative effectors tested showed unique subcellular accumulation patterns with the exception of Las525mp, which was diffused throughout the cytosol and the nucleus (Figures 2A–Q). The different accumulation and localization patterns may be indicative of a specific interaction between the Las protein and plant cell organelles and warrants further investigation in the future. It is important to point out that the Las5315mp effector protein (the mature protein) was localized in vesicles surrounding chloroplast (Figures 2P,Q), while the premature protein did not accumulate in the chloroplast (Figure 2R). These results are rationalized by the fact that the full length protein, Las5315, contains a signal peptide and a chloroplast targeting signal in succession (Figure 6). In an obligate intracellular bacterium such as Las, the signal peptide is assumed to be cleaved by the bacterium upon translocation; leaving only the mature Las5315mp effector protein in the host cell's cytosol. Here, the protein is no longer subjected to this cleavage reaction since it is being produced by the transformed plant not the bacterium and, thus, only the protein without the signal sequence is active.

Plant pathogens secrete effector proteins that are subsequently delivered to the inside of host cells and interfere with their molecular pathways, thereby promoting infection (Dangl and Jones, 2001; Nomura et al., 2005; Hann et al., 2010). These effectors often affect host immunity and cause phenotypes ranging from chlorosis to necrosis (Cunnac et al., 2009). The ability of protein Las5315mp to induce chlorosis and cell death at 3 dpi in *N. benthamiana* in the infiltration zone (Figure 3A) suggests that it may be affecting host immunity, an observation further supported by ion leakage assays (Figure 3B). Plant cell death is a common defense mechanism used by plants against invading pathogens to prevent the spread of the microbial pathogens and can occur in both resistant and susceptible plant–pathogen interactions. Cell death results when the host plant's receptors perceive the pathogen and trigger plant immunity reactions (Zipfel and Felix, 2005; Jones and Dangl, 2006; Takahashi et al., 2007; Coll et al., 2011). Interestingly, cell death has not been detected in citrus in response to Las infection comparable to that seen with *N. benthamiana* infiltrated with Las5315mp, suggesting that although *N. benthamiana* may possess resistance (R) genes, intracellular nucleotide-binding domains, or leucine-rich repeat containing (NB-LRR) immune receptors that specifically recognize Las5315mp effector protein, these NB-LRRs might not be conserved in citrus. Thus, only *N. benthamiana* can generate an effector triggered immunity response and induce cell death upon recognition of the pathogen effector Las5315mp. Indirect support of this is also demonstrated by the fact that previous attempts to infect *Nicotiana tabacum* with Las were unsuccessful (Duan, unpublished data), however *Ca*. L. americanus, which does not have a Las5315mp homolog, was able to infect *N. tabacum* L. cv Xanthi through *Cuscuta* spp (dodder) (Francischini et al., 2007).

Previously, NB-LRR genes have been identified and used to confer broad-spectrum resistance in different crops. Examples include the *WRR4* gene from Arabidopsis that confers white rust resistance in transgenic oilseed brassica crops (Borhan et al., 2010); two other Arabidopsis genes (*RFO1* and *RPW8)* that confer resistance against a diverse collection of Fusarium or powdery mildew fungi (Xiao et al., 2001; Diener and Ausubel, 2005); RCT1 from *Medicago truncatula* that confers resistance to races of the anthracnose fungi *Colletotrichum trifolii* and *C. destructivum* (Yang et al., 2008); the Mi-1.2 gene in tomato *Solanum lycopersicum* that confers resistance to the root-knot nematode *Meloidogyne javanica* in *Solanum melongena* (Goggin et al., 2006); and the *RPS4/RRS1* genes from Arabidopsis that confer resistance to the fungal pathogen *C. higginsianum* to other *brassicaceae* plants including *B. rapa* and *B. napus* as well as in *N. benthamiana* and *Solanum lycopersicum* (Narusaka et al., 2013). In addition, *RGA4 and RGA5* genes from rice mediate resistance to the fungal pathogen *Magnaporthe oryzae* in both rice protoplasts and *N. benthamiana* (Cesari et al., 2014*)*. Therefore, it is quite plausible that identification of the *N. benthamiana* receptor of Las5315mp and its heterologous expression in citrus plants may trigger an innate immune response to the Las pathogen and confer resistance to HLB disease in citrus plants.

**Figure 7:**
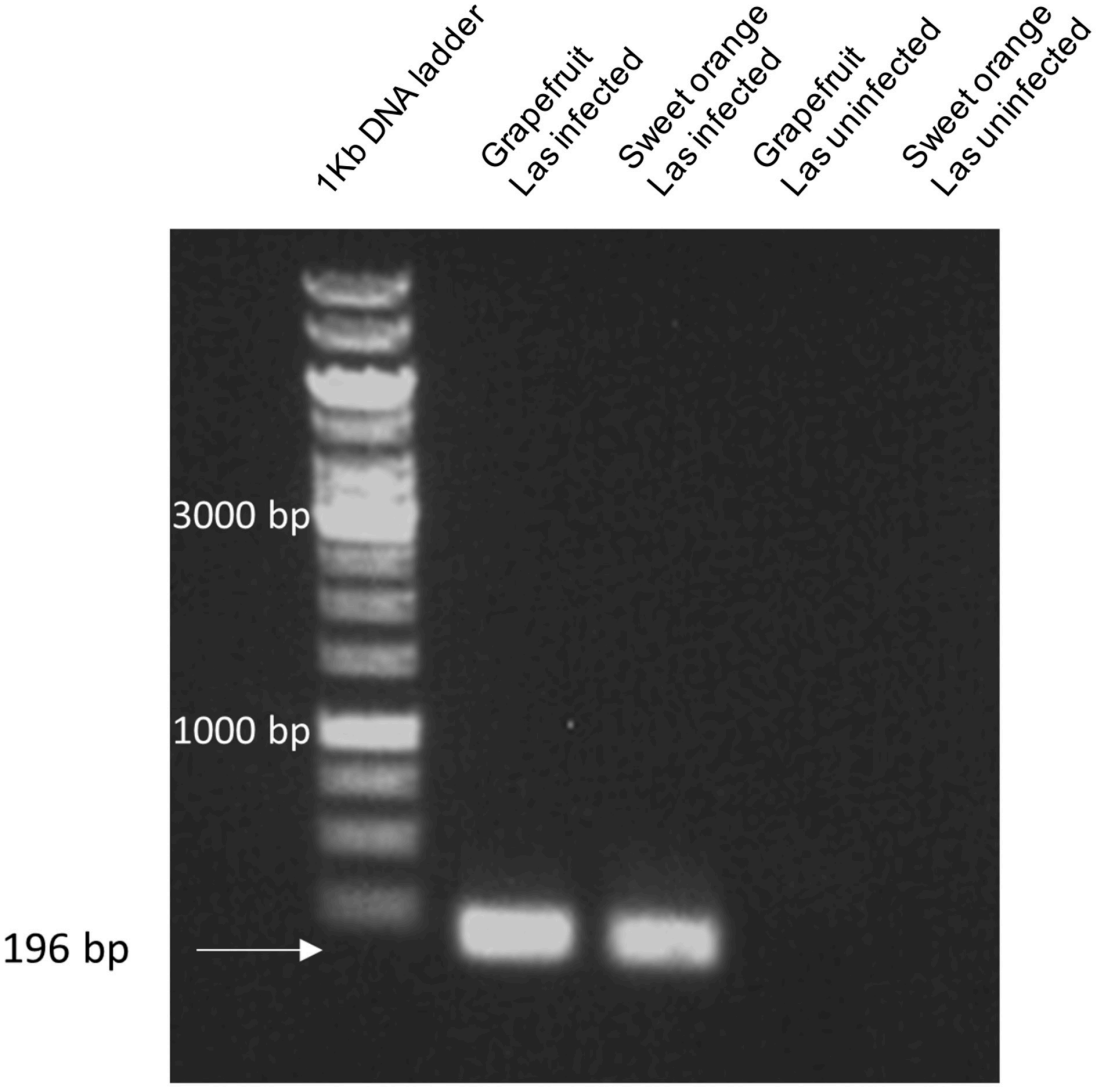
*****Las5315*** expression in citrus using reverse transcription PCR**. Amplicons corresponding to the expression levels of *Las5315* in both Las infected and uninfected grapefruit and sweet orange were produced via reverse transcription PCR. Amplification products were subsequently run on a 1% agarose gel and visualized with ethidium bromide.

The inhibition of photoassimilate export and the subsequent starch accumulation in the Las infected leaves indicates that Las infection perturbs normal carbon partitioning (Koh et al., 2012). Previous studies have demonstrated that Liberibacter infection is accompanied by callose deposition in sieve pores of the sieve tubes and it has been suggested this callose deposition is the main reason for the phloem plugging associated with starch accumulation and ultimately chlorosis of the leaf in infected plants (Masaoka et al., 2011). In addition, genes involved in callose deposition have been shown to be up-regulated in HLB-infected citrus (Koh et al., 2012; Zhong et al., 2015). In this work, we found that Las5315mp induces excessive callose in the infiltration zone starting at 1 dpi (Figure 5) and increases the accumulation of callose in the cells and inside the vascular tissue in a fashion similar to that seen in citrus plants infected by Las (Koh et al., 2012). Since *Las5315* is also expressed in HLB infected citrus (Figure 7), it stands to reason that Las5315mp may be the protein associated with callose deposition in HLB affected citrus. Further functional characterization and identification of Las effectors associated with pathogenicity and virulence should lead to identification of potential targets for controlling Las infection and HLB disease progression.

## Author contributions

Conceived and designed the experiments: MP, YD. Performed the experiments: MP. Analyzed the data: MP, CA, LC. Contributed reagents/materials/analysis tools: MP, CA, YD. Wrote the paper: MP, CA, YD.

### Conflict of interest statement

The authors declare that the research was conducted in the absence of any commercial or financial relationships that could be construed as a potential conflict of interest. The reviewer WU declared a shared affiliation, though no other collaboration, with several of the authors MP, CA, YD to the handling Editor, who ensured that the process nevertheless met the standards of a fair and objective review.

## Supplementary material

Figure S1 **Subcellular localization of putative Las effector proteins in *Nicotiana benthamiana***. Sixteen putative effector proteins expressed in leaves of *N. benthamiana*, visualized by epifluorescence microscopy 3 days post infiltration. **(A–O)** Micrograph of the localization of the 15 candidate effectors that did not elicit a phenotype in *N. benthamiana*. **(P)** Image shows localization of Las5315mp:GFP (green) surrounding chloroplasts (red). **(Q)** Enlarged image of panel **(P)** with arrows indicating Las5315mp localization surrounding chloroplasts. **(R)** Las5315::GFP accumulated in aggregate structures **(S)** GFP only. Scale bars are 20 μm.

Figure S2 **Effector Las5315mp induces cell death in *N. benthamiana***. Phenotype of *N. benthamiana* leaves expressing **(A)** Las5315mp or **(B)** Las5315 at several different concentrations (OD_600_ = 0.1, 0.3, 0.5) compared to the empty vector control. **(C)** Overexpression of Las5315mp and Las5315 in the same leaf 7 days post infiltration (dpi) at an OD_600_ = 0.5.

Table S1 **Primers used in this study**.
