# Supplementary figures and images for: Transient Expression of Candidatus Liberibacter Asiaticus Effector Induces Cell Death in Nicotiana benthamiana
Source: Front Plant Sci. 2016 Jul 6;7:982. doi: 10.3389/fpls.2016.00982 (PMC4933711; doi:10.3389/fpls.2016.00982)

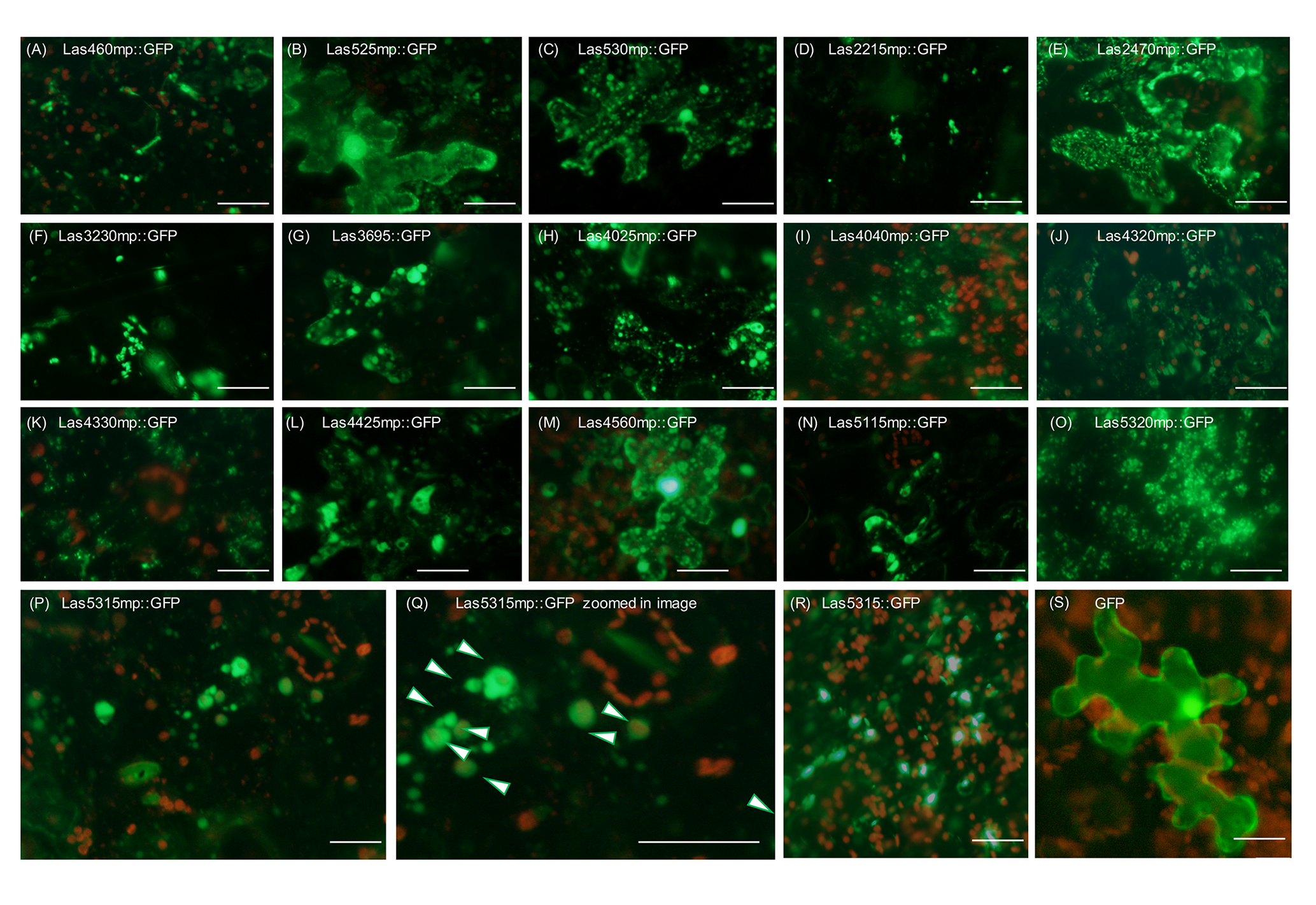

Supplement: Figure S1 — Subcellular localization of putative Las effector proteins in Nicotiana benthamiana. Sixteen putative effector proteins expressed in leaves of N. benthamiana, visualized by epifluorescence microscopy 3 days post infiltration. (A–O) Micrograph of the localization of the 15 candidate effectors that did not elicit a phenotype in N. benthamiana. (P) Image shows localization of Las5315mp:GFP (green) surrounding chloroplasts (red). (Q) Enlarged image of panel (P) with arrows indicating Las5315mp localization surrounding chloroplasts. (R) Las5315::GFP accumulated in aggregate structures (S) GFP only. Scale bars are 20 μm. [file Image1.tif]

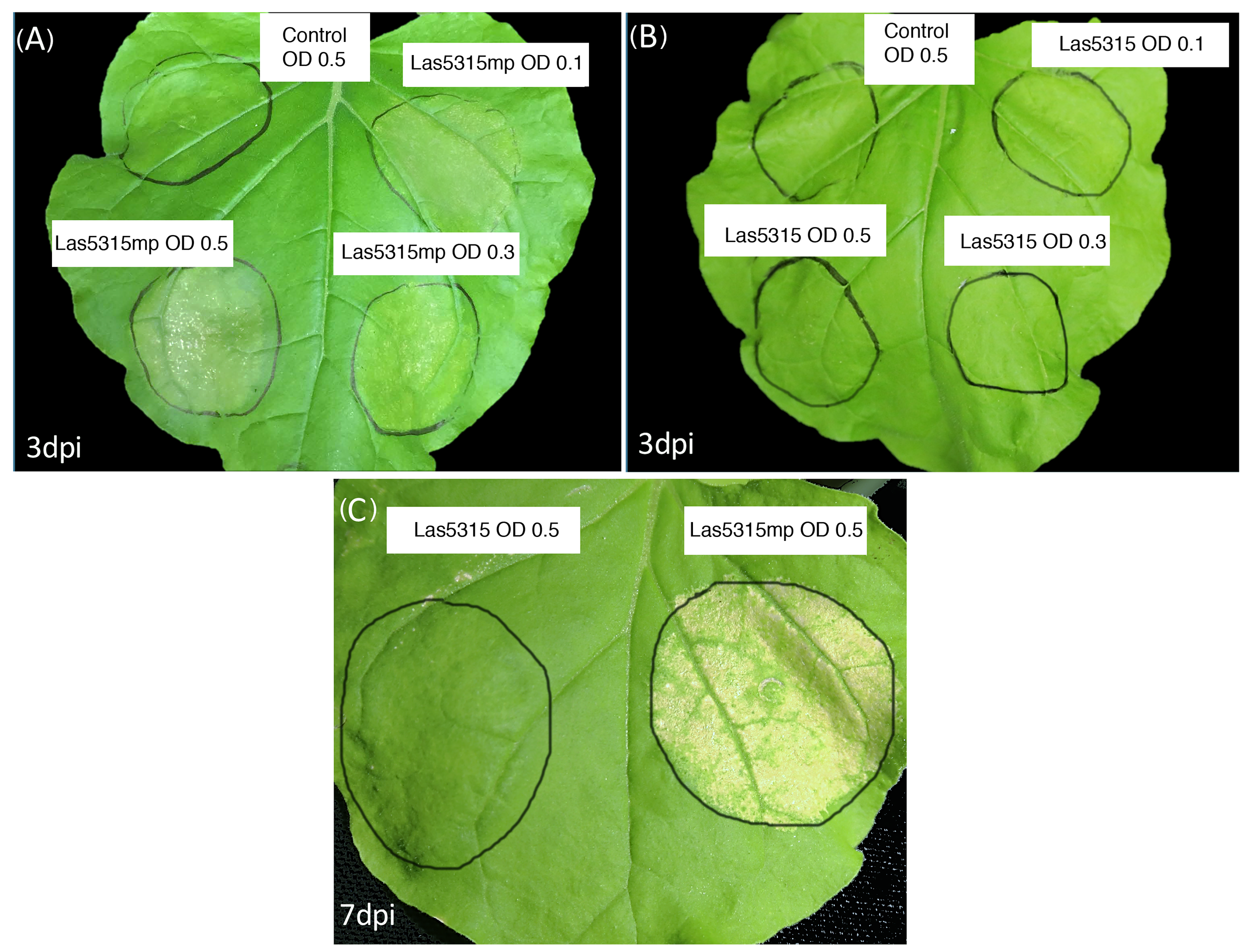

Supplement: Figure S2 — Effector Las5315mp induces cell death in N. benthamiana. Phenotype of N. benthamiana leaves expressing (A) Las5315mp or (B) Las5315 at several different concentrations (OD600 = 0.1, 0.3, 0.5) compared to the empty vector control. (C) Overexpression of Las5315mp and Las5315 in the same leaf 7 days post infiltration (dpi) at an OD600 = 0.5. [file Image2.TIF]
